# Interventions to improve or facilitate linkage to or retention in pre-ART (HIV) care and initiation of ART in low- and middle-income settings – a systematic review

**DOI:** 10.7448/IAS.17.1.19032

**Published:** 2014-08-01

**Authors:** Darshini Govindasamy, Jamilah Meghij, Eyerusalem Kebede Negussi, Rachel Clare Baggaley, Nathan Ford, Katharina Kranzer

**Affiliations:** 1 Health Economics and Epidemiology Research Office (HE^2^RO), WITS Health Consortium, University of the Witwatersrand, Johannesburg, South Africa; 2 Malawi Liverpool Wellcome Trust Clinical Research Programme, Blantyre, Malawi; 3 HIV/AIDS Department, World Health Organization, Geneva, Switzerland; 4 Department of Infectious Disease Epidemiology, London School of Hygiene and Tropical Medicine, London, United Kingdom

**Keywords:** HIV, antiretroviral therapy, pre-ART, ART eligibility, ART initiation, retention in care, linkage to care, attrition, low- and middle-income countries

## Abstract

**Introduction:**

Several approaches have been taken to reduce pre-antiretroviral therapy (ART) losses between HIV testing and ART initiation in low- and middle-income countries, but a systematic assessment of the evidence has not yet been undertaken. The aim of this systematic review is to assess the potential for interventions to improve or facilitate linkage to or retention in pre-ART care and initiation of ART in low- and middle-income settings.

**Methods:**

An electronic search was conducted on Medline, Embase, Global Health, Web of Science and conference databases to identify studies describing interventions aimed at improving linkage to or retention in pre-ART care or initiation of ART. Additional searches were conducted to identify on-going trials on this topic, and experts in the field were contacted. An assessment of the risk of bias was conducted. Interventions were categorized according to key domains in the existing literature.

**Results:**

A total of 11,129 potentially relevant citations were identified, of which 24 were eligible for inclusion, with the majority (*n*=21) from sub-Saharan Africa. In addition, 15 on-going trials were identified. The most common interventions described under key domains included: health system interventions (i.e. integration in the setting of antenatal care); patient convenience and accessibility (i.e. point-of-care CD4 count (POC) testing with immediate results, home-based ART initiation); behaviour interventions and peer support (i.e. improved communication, patient referral and education) and incentives (i.e. food support). Several interventions showed favourable outcomes: integration of care and peer supporters increased enrolment into HIV care, medical incentives increased pre-ART retention, POC CD4 testing and food incentives increased completion of ART eligibility screening and ART initiation. Most studies focused on the general adult patient population or pregnant women. The majority of published studies were observational cohort studies, subject to an unclear risk of bias.

**Conclusions:**

Findings suggest that streamlining services to minimize patient visits, providing adequate medical and peer support, and providing incentives may decrease attrition, but the quality of the current evidence base is low. Few studies have investigated combined interventions, or assessed the impact of interventions across the HIV cascade. Results from on-going trials investigating POC CD4 count testing, patient navigation, rapid ART initiation and mobile phone technology may fill the quality of evidence gap. Further high-quality studies on key population groups are required, with interventions informed by previously reported barriers to care.

## Introduction

The rapid roll-out of antiretroviral therapy (ART) programmes over the past decade in low- and middle-income countries has resulted in an estimated 9.7 million HIV-positive individuals receiving ART by the end of 2012, compared to just 300,000 in 2000 [1]. HIV testing and counselling, the entry point to care, has also been brought to scale at an impressive pace [1]. However, overall programmatic success has not reached its full potential due in part to the high attrition occurring in the period between HIV testing and ART initiation. Mathematical modelling studies have shown that achieving the maximum population-level prevention benefits from the proposed universal test and treat strategies is dependent on successful programmatic outcomes, including optimum linkage to care rates [2–4]. It is estimated that only 65% of those eligible for treatment as of the end of 2012 were currently receiving ART [1,5,6], and by raising the ART initiation threshold to CD4<500 cells/µL as per World Health Organization (WHO) guidelines released in July 2013, coverage is estimated to be considerably lower [1]. If major progress towards universal access to ART is to be attained by 2015 then there is an urgent need for HIV programmes to strengthen the existing HIV continuum of care pathway and ensure that increased access to HIV testing is accompanied by improved linkage to care.

To date, attempts have been made to quantify the losses occurring along the HIV cascade and describe the barriers to care, although data from low- and middle-income countries outside sub-Saharan Africa are scarce. Three recent systematic reviews have consistently shown major attrition occurring along each step of the HIV cascade in sub-Saharan Africa for adults. Less than half (45–46%) of individuals not yet eligible for ART were retained in pre-ART care and only two-thirds (65–68%) of ART-eligible individuals initiated ART [7–9]. A recent HIV cascade analysis conducted in India found that approximately 70% of HIV-positive patients linked to care within three months post-diagnosis, with only 65% of pre-ART patients retained in early care and 67% of ART-eligible patients who initiated ART within three months [10]. A systematic review investigating patient and programme level factors associated with retention in care during the pre-ART period and linkage to ART care in sub-Saharan Africa found that the commonly cited barriers included psychosocial (stigma and fear of disclosure), economic (inability to afford transport costs, distance to healthcare facility) and health system (long waiting times, shortage of health care workers) factors [11]. Moreover, poor linkage to care has been reported among groups such as pregnant women [12] and children [13]. Among ART-eligible pregnant women in low- and middle-income countries, 38–88% failed to initiate ART, though point of attrition assessed by the different studies differ, with financial constraints and fear of stigma identified as the main obstacles to ART care [12]. High rates of pre-ART mortality and loss to follow-up prior to starting ART among children have also been reported in studies from sub-Saharan Africa [13]. According to the new ART eligibility criteria recommended by the WHO guidelines, the number of HIV-positive individuals deemed eligible for ART will increase considerably; however, it will decrease the time period from entry to care and ART eligibility and likely reduce attrition in the pre-ART period [1,14].

Patients in the period between HIV diagnosis and ART initiation can be divided into three groups: those that enter into HIV care post-diagnosis, those that remain in pre-ART care until ART-eligible, and those that initiate ART once ART eligibility is established. Rosen et al. describe these three periods as stage 1, 2 and 3, respectively [12]. Several interventions to improve ART adherence and retention have been rigorously evaluated [15], including treatment supporters [16], nutritional support [17–19], mobile-text messages [20]. However, research on interventions to increase linkage to and retention in pre-ART care and initiation of ART has only recently emerged. Interventions that have been tested include point-of-care (POC) CD4 count testing [21–23], referral slips [24], patient navigators [25], home-based HIV testing and ART initiation [26,27]. The aim of this review is to assess the effect of interventions to improve or facilitate linkage to or retention in pre-ART care and initiation of ART in low- and middle-income settings.

## Methods

Randomized trials, comparative non-randomized observational studies, and comparative before-and-after studies were eligible for this review. Cohort studies were only included if the control (comparator) group was contemporary (delivered at the same time and in the same country). Studies were eligible if they included HIV-positive patients in low- and middle-income countries (as defined by the World Bank [28,29]) at any point before initiating treatment and described an intervention aimed at improving linkage or retention in pre-ART care or initiation of ART. Primary outcomes were defined as proportion of HIV-positive patients 1) retained in pre-ART care, 2) linked to care and 3) initiated on ART as defined by study author and 4) time to linkage or initiation of ART. Secondary outcome assessed were: 1) patient satisfaction with care, as defined by the study authors 2) cost to the provider and 3) cost to the patient and family.

The literature was searched for available systematic and narrative reviews investigating linkage to pre-ART, HIV and ART care and retention in pre-ART care. Five systematic reviews were identified [7–10,30] and the reference lists were hand searched. Additional searches were conducted in four electronic databases for primary studies: Medline, Embase, Global Health and Web of Science from the 1st of January 2004 (start of ART delivery at scale in low- and middle-income countries) to the 10th of February 2013. The detailed compound search strategies are described in Supplementary Tables 1–3. Three electronic databases were searched for on-going trials: Cochrane Central Register of Controlled Trials (CENTRAL), WHO International Clinical Trial Registry Platform and ClinicalTrials.gov. Furthermore, a total of 117 experts (Supplementary Tables 4 and 5) were contacted to identify additional studies either completed or on-going; these experts were authors of studies included in this and previous reviews [7–10] and these experts were asked to provide additional names of people who were subsequently contacted in a second round. Finally, reference lists of all studies identified by the above methods were screened for potentially eligible studies. Conference abstract databases of the following conferences were searched: Conferences on HIV Pathogenesis and Treatment of the International AIDS Society (IAS, 2001–2011), Conferences on Retroviruses and Opportunistic Infections (CROI, 2006–2013) and the International AIDS Conferences (AIDS, 2001–2012). Additional searches were undertaken to identify subsequent publications of studies presented at conferences. These searches were conducted in Ovid Medline using the first and the last authors’ names combined with “HIV” and the country the study was conducted in. Authors of publications with potentially relevant data were contacted in case clarification was needed.

All references identified by the compound search strategy were imported into EndNote. After duplicates were removed, titles and abstracts were examined independently by the two authors (DG, KK). The full text of potentially relevant studies was obtained and the inclusion criteria were applied independently using a standardized eligibility form. Data extraction was performed by KK using a standardized data extraction form. Information was extracted regarding study (citation, start and end dates, location, study design and details), participants (eligibility criteria, age range, gender, population size, specific high-risk groups, HIV disease progression), intervention (detailed description of the intervention and standard of care in the control groups) and outcomes (proportion of individuals with a positive outcome and time to accessing HIV or ART care). Drawing on knowledge from previous systematic reviews of risk factors and barriers to care, interventions were categorized as follows: health system interventions, patient convenience and accessibility, behaviour interventions and peer support, and incentives. In addition, the stage the intervention was targeted at in the HIV cascade was also highlighted. The risk of bias was assessed by the Cochrane Effective Practice and Organisation of Care (EPOC) Group criteria for randomized-controlled and non-randomized-controlled trials [31] and the Newcastle-Ottawa Scale for observational studies [32].

## Results

### Study characteristics

**Figure 1:**
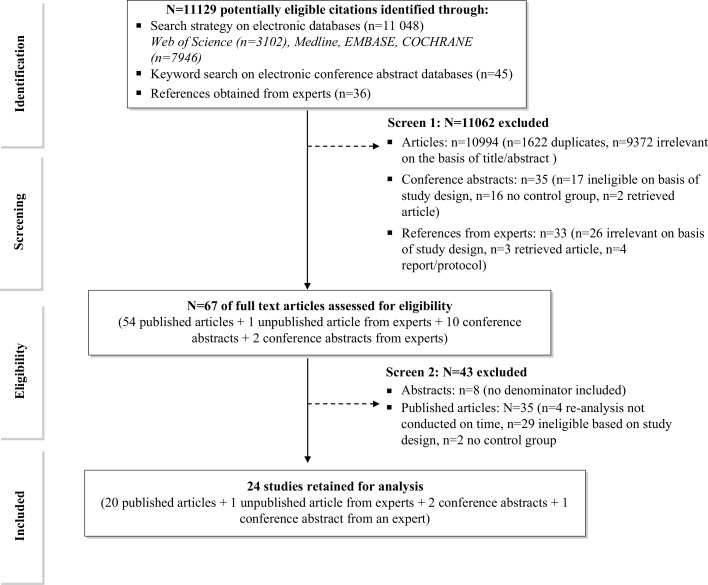
Selection process for the inclusion of studies.

A total of 11,129 potentially relevant citations were identified, of which 24 were eligible for inclusion (Figure 1). In addition, 15 on-going trials investigating interventions to improve linkage and retention in pre-ART care were identified. Four publications with potentially relevant data could not be included as authors were unable to provide relevant data [18,24,33,34]. Characteristics of the included studies are summarized in Tables 1 and 2. Twenty studies were published in peer reviewed journals, one was an unpublished study and three were conference abstracts. There were 21 studies from sub-Saharan Africa (nine countries), two studies from India [35,36] and one study from Cambodia [37]. The following patient subgroups were included: patients co-infected with tuberculosis (TB) (*n*=1) [37], children (*n*=1) [35], adolescents (*n*=1) [38], inpatients (*n*=1) [39], injecting drug users (*n*=1) [36] and pregnant women (*n*=9) [40–48]. No studies were identified that investigated interventions in any other key population group (men who have sex with men, sex workers, the elderly, prisoners and migrants). There were no studies identified from Central Asia, the Americas or the Pacific region.

**Table 1:** Description of full-text studies included in the review (listed alphabetically by country)

Author	Year of study	Country	Setting	ART eligibility criteria	Study design	Intervention and control	Outcome measures	Comments
Choun [37]	2008–2011	Cambodia	Phnom Penh, 1 hospital (outpatient), TB patients	2008–2010: extrapulmonary TB, pulmonary TB and CD4 <350 cells/µL 2010–2011: All TB patients	Before/after study	*Intervention* Communication Weekly medical department meeting (TB and HIV physicians) Monthly multidisciplinary meeting of key HIV and TB care staff to discuss logistical, managerial, operational issues Daily educational activities for HIV patients attending the HIV clinic Preparation of implementation Required changes in patient flow discussed: key changes consisted of more rapid pre-ART counselling and fast-tracking for ART initiation No concurrent changes in TB case-finding strategies or TB and HIV care clinic organizational set-up (staffing, HIV/TB service delivery model) Fixed starting date of implementation Monitoring Within each service, supervisors monitored adherence to the implementation during their routine (clinical) work as part of their continuous monitoring of all guidelines and practices Operational challenges with implementation were raised by staff meetings	Proportion of individuals starting ART within 4 or 8 weeks of TB diagnosis Time from TB diagnosis to ART initiation	Age and sex were similar in control and intervention group CD4 (cells/µL) median (IQR): Control: 59 (18–168) Intervention: 43 (20–163) No outcome verification Adjustment for confounding (CD4 count, extrapulmonary TB, enrolment in HIV care before TB diagnosis)
Kundu [35]	2008–2009	India	Kolkata (urban), 1 tertiary hospital (outpatients), children	Not stated	Before/after study	*Control (SOC):* Caregivers were advised to follow-up in the paediatric pre-ART clinic at least once in 2 months. If children failed to visit the clinic >2 months, then guardians were contacted by phone call *Intervention:* Provision of supplementary nutrition as monthly take home rations The rations were distributed outside the hospital premises on the principle of providing one-third calorie and one-half proteins of recommended daily allowance for the age of the child	Proportion of children with regular pre-ART follow-up (>6 visits per year) over a 12 months period Proportion of children remaining in care (children with an appointment within the last 6 months) over a 12-month period	Potential for survival bias (3 children died, 6 children were lost to follow-up and 11 children started ART during the first year (control)) No adjustment for confounding
Hatcher [25]	2009	Kenya	Nyanza (rural) community-based testing, adults	CD4 cell count <250 cells/µL	Observational study with control group	*Control:* No home follow-up *Intervention:* Follow-up home visit by a trained PLHA navigator	Proportion of individuals enrolled at a clinic 10 months after the positive HIV test	Only individuals who consented to a home visit were assigned the intervention and only individuals who were located received the intervention Outcomes were only ascertained for individuals traced and found 10 months after the testing No outcome verification, ascertainment of outcome through self-report Adjustment for confounding (age, education, ART knowledge, discrimination, disclosure health status, distance to HIV clinic)
Kohler [50]	2005–2007	Kenya	Nairobi (urban), 1 outpatient site in a secondary hospital	CD4 cell count <250 cells/µL or WHO stage 3 or 4	Before/after study	*Control:* 6 monthly health care visits for patients not yet eligible for ART, follow-up through phone calls for patients with missed appointments *Intervention:* Free co-trimoxazole, scheduled monthly visits for refill, follow-up through phone calls for patients with missed appointments	Proportion of individuals not yet eligible for ART in care 12 months after enrolment	Age, sex and BMI were similar in the intervention and control group CD4 (cells/µL) median: Control: 412 Intervention: 441 Adjustment for confounding (age, sex, CD4 count)
MacPherson [26]	2012	Malawi	Northwest Blantyre (urban), community, adult residents (>16 years of age)	CD4 cell count <350 cells/µL, WHO stage 3 or 4, pregnant or breastfeeding	Cluster randomized trial	*Control:* HIV self-testing kits were available for all cluster residents through community counsellors. All individuals who requested self-testing could do so in the privacy of their home. They were asked to return the used kit, but were told that they were not required to disclose the result, but if they wish to do so they would receive post-test counselling. All self-testing participants received a self-referral card to access HIV care (and ART if eligible) at local health facilities *Intervention:* Self-testing and facility-based HIV care was available for all community members as in the control clusters. Individuals who disclosed a positive result to the community counsellor could opt to receive home-based initiation of HIV care. If requested a study nurse would undertake a home visit within 3 days and perform confirmatory testing, ART eligibility assessment (CD4 count, WHO stage), undertake TB screening and provide INH and the first of the two treatment preparedness sessions. At a second visit individuals who met national eligibility criteria received 2 weeks of ART before transfer into the routine clinic system.	Proportion of individuals initiated on ART among adult cluster residents	Age and sex was equally balanced in intervention and control clusters No outcome verification Outcome was assessed blinded to the intervention Adjustment for clustering
Weigel [41]	2006–2009	Malawi	Lilongwe (rural), 1 antenatal care clinic, pregnant women	CD4 cell count <250 cells/µL or WHO stage 3 or 4	Before/after study	*Intervention* July 2006: CD4 testing at 1st ANC visit during provider initiated testing and counselling, introduction of referral slip Sept 2006: Vehicle for transport of women from ANC to ART clinic, Health passports provided free to pregnant women Dec 2006:New ART centre opens 200 m walking distance from ANC clinic; identification of ART clinic staff member acting as a link person to ANC and being responsible for ‘PMTCT women’ May 2007: No queuing for pregnant women, no guardian required to start ART on the same day Mar 2008: introduction of leaflets and poster signposts to mark the way from ANC to ART Dec 2008: introduction and use of an electronic data system with a unique hospital number Oct 2009: New ANC and maternity opens opposite of ART site	Proportion of pregnant women who registered with the ART clinic if they had a CD4 count Proportion of pregnant women who initiated ART among the ones who had registered	Age and CD4 counts similar across years Small sample size in the initial year (2006) Outcomes were not time delineated (maximum and minimum follow-up time unknown) No adjustment for confounding
Jani [21]	2009–2010	Mozambique	Maputo and Sofala (rural and per-urban), 4 primary health care clinic, 1 year+	>15 years: CD4 cell count <250 cells/µL 4–15 years: <350 cells/µL 1–4 years: <750 cells/µL	Before/after study	*Control:* Blood samples for CD4 counts were collected once a week and sent to a nearby laboratory, patients were asked to return for a staging visit after the test results were available *Intervention:* POC CD4 count testing was done on the same day in the phlebotomy room where a laboratory assistant tested fingerpick samples Patients with results were referred to consultation rooms to be staged and to have eligibility assessed on the same day	Proportion of individuals who completed a staging visit (receipt of CD4 result and assessment of ART eligibility) within 90 days of testing Proportion of individuals initiating ART if found eligible within 60 days of testing Days between CD4 result and ART initiation for participants found eligible Proportion of individuals initiating ART within 150 days of testing Days between testing and ART initiation	Age and sex were similar in control and intervention group No outcome verification (outcome was ascertained through folder review) Adjustment for confounding (age, sex, clinic)
Pfeiffer [48]	2004–2007	Mozambique	Manica and Sofala districts (rural and urban)	Not stated	Observational study with control group	*Control:* vertical, donor-initiated, day hospital (=HIV treatment hospitals in large population centres alongside existing hospital compounds) *Intervention:* decentralization and integration of HIV-related programmes into primary health care services: 1) co-location of different services within the same facility 2) training of personnel to provide multiple services, 3) provision of tools, processes and training to better link services 4) strengthening of linkages, referral and follow-up between facility levels 5) harmonization of logistic systems	Proportion of eligible individuals initiating ART within 90 days Proportion of HIV-positive pregnant women registered for HIV care within 30 days of testing	No data on denominators presented No baseline data No outcome verification (routine data collection) No adjustment
Tsague [40]	2008	Rwanda	National, pregnant women	No stated	Observational study with control group	*Control* (“stand-alone”): only PMTCT services were available at the ANC site *Intervention* (“full package”): PMTCT and ART services were both available on site	Proportion of HIV-positive pregnant women undergoing a CD4 cell count assessment during pregnancy Proportion of HIV-positive pregnant women enrolling into HIV care and treatment during pregnancy Proportion of eligible HIV-positive pregnant women initiating ART during pregnancy	No outcome verification No baseline data (routine data collection) No adjustment, no controlling for clustering
Larson [23]	2010	South Africa	Johannesburg, 2 mobile clinics, adults	CD4 cell count <200 cells/µL or WHO stage 4 disease	Observational study with control group	*Control (SOC)*: patients with a positive HIV test result received post-test counselling and referral to an HIV care and treatment site *Intervention*: SOC POC CD4 count testing	Proportion of individuals enrolling in HIV care within 8 weeks of HIV testing	Mean age was similar in control and intervention group. There were more women (62%) in the intervention group compared to the control group (55%) Outcome was only ascertained in 63% No outcome verification, outcome ascertainment through self-report Adjustment for age, sex, previous HIV testing experience
Faal [22]	2009	South Africa	Johannesburg (urban), 1 primary health care clinic, adults	CD4 cell count <200 cells/µL or WHO stage 4 disease	Individual randomized controlled trial	*Control A*: blood for CD4 count was drawn at the time of testing, patients were required to return to receive the result *Control B*: blood for CD4 count was drawn at the time of testing, patients were required to return to receive the result, but they received a leaflet with information *Intervention:* blood for CD4 count was drawn at the time of testing and the patient received the result on the same day	Proportion of individuals reporting for further care within 1 month of HIV testing if not yet eligible (CD4 count >215 cells/µL) Proportion of individuals initiating ART within 3 months of HIV testing if eligible (CD4 count <215 cells/µL)	Baseline variables were distributed equally across the groups No outcome verification
Fairall [51]	2008–2010	South Africa	Free State (urban and rural), 31 primary health care clinics, adults (>16 years of age)	Non-pregnant women and men: CD4 cell count <200 cells/µL or WHO stage 4 disease Pregnant women: CD4 cell count <350 cells/µL	Cluster randomized control trial	*Control:* Patients diagnosed with HIV were referred to designated nurse-led clinics to establish whether they were eligible for ART. Patients not yet eligible for ART received routine care, such as regular CD4 testing, until they became eligible. Patients eligible for ART were referred to ART treatment sites in hospital outpatient departments for initiation of treatment and review of ART prescriptions every 3–6 months, done by a doctor *Intervention:* Prescribing nurses received at least four educational outreach training sessions about ART prescribing and side-effects with a special edition of the PALSA PLUS guidelines, which included algorithms to start and to monitor patients on ART, and to identify those needing referral to a doctor. Patients had to meet certain criteria for nurse initiation and re-prescription of ART. Patients who did not meet these criteria were referred to doctors who, unlike nurses, were authorized to initiate tailored regimens, to change prescriptions, and to prescribe second-line drugs	Proportion of individuals in HIV care (either pre-ART care or ART care) 6 months of enrolment visit among individuals with CD4 count <350 cell/µL	Age and sex was similar in intervention and control clusters CD4 (cells/µL) median (IQR): Control: 158 (78–232) Intervention: 163 (84–241) No outcome verification Ad-hoc sub-analysis (not the primary endpoint of the trial) Adjustment for clustering
Youngleson [44]	2006–2009	South Africa	Western Cape (urban and rural), 17 antenatal care clinics, pregnant women	CD4 cell count <200 cells/µL or WHO stage 4 disease	Before/after study	*Control:* Stand-alone ANC clinic *Intervention:* Resource addition and process improvement Six learning workshops between 2006–2009 for facility staff and managers Additional HAART clinic at primary care site with large antenatal clinic and high HIV prevalence Improved communication between ANC and ART clinic, antenatal clients were walked over to ART room ‘Mother’s Day’: dedicated ART clinic day each week for pregnant women needing ART Mother2mothers program: support and education to women in PMTCT program	Proportion of pregnant women who were receiving ART at time of delivery among all women testing HIV positive	No information on age and CD4 count No outcome verification No adjustment for confounding
Stinson [45]	2005	South Africa	Cape Town, 3 antenatal care clinics, pregnant women	CD4 cell count <200 cells/µL or WHO stage 4 disease	Observational study with control group	*Control:* eligible women were referred to an ART clinic within 5 km *Proximal service:* eligible women were referred to an ART clinic, which was on the same premises, but not in the same building *Integrated service:* outreach doctors came to the ANC clinic twice a week	Proportion of ART eligible pregnant women initiated on ART during pregnancy	Age was similar in all three groups CD4 (cells/µL) median (IQR): Control: 139 (106–172) Proximal: 135 (94–167) Integration: 143 (97–171) Only three clinics were included No outcome verification Adjustment for age and gestational age
Van der Merwe [46]	2004–2005	South Africa	Gauteng (urban), 1 hospital, outpatient, antenatal care, pregnant women	CD4 cell count <200 cells/µL or WHO stage 4 disease	Before/after study	*Control:* Women were referred to an off-site ART clinic *Intervention:* ART clinician came to the ANC clinic once a week CD4 count were performed at the first ANC visit Women received the CD4 count at the second ANC visit and had baseline bloods if CD count <250 cells/µL For women eligible for ART, adherence counselling and treatment preparation was scheduled for the 2nd visit	Time from eligibility assessment to initiation of ART for pregnant women eligible for ART	Age was similar in all three groups CD4 (cells/µL) median (IQR): Control: 146 (117–178) Intervention: 143 (92–186) No outcome verification No adjustment for confounding
Burtle [49]	2009–2010	Swaziland	Lumbombo (rural), 1 secondary hospital, all patients (excluding pregnant women and patients with TB)	CD4 cell count <200 cell/µL or WHO stage 4	Before/after study	*Control:* HIV care prior to commencing ART was episodic *Intervention:* 1) comprehensive care using a patient care pathway (with three records inpatient pre-ART file, patient hand-held file, pre-ART registration book), 2) active follow-up by cell phone and adherence officers 3) task shifting to nurses and lay counsellors	Proportion of individuals with documents assessment of eligibility Proportion of ART eligible individuals initiated on ART Days between eligibility assessment and ART initiation	Age and sex were similar in baseline group, during implementation of the intervention, one year after implementing the intervention CD4 (cells/µL) median (range): Baseline: 281 (2–2003) Implementation: 292 (2–2777) Post implementation: 241 (4–1815) No outcome verification Outcomes were not time delineated, the minimum and maximum time of follow-up was 3 and 15 months
Muhamadi [52]	2009–2010	Uganda	Iganga (rural), 1 outpatient site in a secondary hospital, 2 primary health care centres,	Not stated	Individual randomized controlled trial	*Control:* Post-test counselling by staff not trained in basic counselling skills and included declaration of the results, provision of co-trimoxazole prophylaxis and advice to the client to go for pre-ARV care every three months *Intervention:* Specialized counselling by health care workers who had been trained on basic counselling skills for 3 days. The counselling included declaration of the results, provision of co-trimoxazole prophylaxis and advice to the client to go for pre-ARV every three months plus encouragement of disclosure of the HIV status, HIV prevention and emphasized the importance of going to pre-ART care. In addition the participants were attached to	Proportion of individuals enrolled in pre-ART care 5 months after testing	Age and sex were similar in control and intervention group CD4 count was not measured No outcome verification, outcome ascertainment through self-report Adjustment for several variables (recruitment centre, age, sex, marital status) an HIV/AIDS community support agent close to their area of resident who visited them monthly at their homes for a two hour counselling session and reminded them to go for quarterly pre-ARV care		
Wanyenze [39]	2004–2005	Uganda	Kampala (urban), 1 tertiary hospital, adult inpatients	CD4 cell count <200 cells/µL or WHO stage 4 (but free ARV not widely available)	Individual randomized controlled trial	*Control:* Testing as an outpatient (a referral card and an appointment to return to the hospital 1 week after discharge was provided, transport was reimbursed) *Intervention:* Testing as an inpatient (testing was conducted within the medical ward and the patient underwent phlebotomy and serological testing, the results were disclosed the following day)	Proportion of individuals attending an HIV clinic within 6 months of testing for those testing positive	Age and sex were similar in control and intervention group CD4 count was not measured No outcome verification, outcome ascertainment through self-report The analysis used the number of participants testing positive as denominator, not the number of participants enrolled in each group
Topp [53]	2008–2011	Zambia	Lusaka (urban), 7 primary health care clinics with voluntary testing and counselling, provider initiated testing and counselling was introduced in a step wise approach	CD4 cell count <200 cells/µL or WHO stage 3 or 4	Observational study with control group	*Control (VCT):* Staffed by nurses whose primary responsibility lay elsewhere, but they provided VCT in a spare room in their free time. The service was available on an ad hoc basis. The majority of clients were self-initiated and counselling was conducted according to psychosocial principles without reference to clients’ clinical condition. If a client tested positive they received a referral slip and required to self-present to the HIV care department *Intervention (PITC):* PITC was offered routinely on an opt-out basis to all clients attending the OPD. PITC was located within the OPD and was part of the patient flow (no separate queuing). PITC was offered by lay personnel and thus only available Monday-Friday 5 hours per day	Proportion of ART eligible individuals initiated on ART Proportion retained in care at 6 months (including pre-ART and ART care)	Proportion retained in care at 6 months (including pre-ART and ART care) CD4 (cells/µL) median (IQR): Control: 224 (110–387) Intervention: 264 (137–427) One of the outcomes (proportion initiating ART) was not time delineated, the minimum and maximum time of follow-up was 1 and 36 months No outcome verification Adjustment for confounding and clustering (age, sex, CD4 count, TB, BMI, education, HIV status)
Killam [47]	2007–2008	Zambia	Lusaka, 8 primary health care clinics, antenatal care, pregnant women	CD4 cell count <350 cells/µL or WHO stage 3 or 4	Before/after study	*Control:* ART clinic located on the same premises as the ANC clinic, but physically separate and separately staffed *Intervention:* ART provision in the ANC clinic, ART services were provided 1–2 days per week	Proportion of ART eligible pregnant women enrolling in ART care within 60 days of testing and before estimated date of delivery Proportion of ART eligible pregnant women initiated on ART within 60 days of testing and before estimated date of delivery	Age was similar in control and intervention group CD4 (cells/µL) mean (SD): Control: 161 (61) Intervention: 166 (58) Adjustment for clinic clustering
Patten [38]	2010–2011	South Africa	Cape Town, primary healthcare clinic, youth	Before August 2011: CD4 cell count <250 cells/µL or WHO stage 3 or 4 After August 2011: CD4 cell count <350 cells/µL or WHO stage 3 or 4	Before/after study	*Control:* blood for laboratory CD4 count was drawn at on the day of an HIV-positive diagnosis and, patients were required to return to receive the result *Intervention:* POC CD4 count testing and notification of results were done on the same day of an HIV –positive diagnosis	Assessment of ART eligibility Proportion of ART eligible HIV-positive youth initiated on ART Time to ART initiation	Age was similar in control and intervention group. There were significant difference in sex between the “control” and the “intervention” group CD4 (cells/µL) median (IQR): Control: 355 (242–491) Intervention: 414 (316–548) No outcome verification. One of the outcomes (proportion initiating ART) was not time delineated No adjustment for confounding

ART=antiretroviral therapy; TB=tuberculosis; IQR=interquartile range; SOC=standard of care; PLHIV=person living with HIV/AIDS; BMI=body mass index; WHO=World Health Organization; INH=isoniazid; ANC=antenatal care; PMTCT=prevention of mother to child transmission, POC=point-of-care; PALSA PLUS=Practical Approach to Lung Health in high-HIV prevalence countries; HAART=highly active antiretroviral therapy; VCT=voluntary counselling and testing; PITC=provider initiated testing and counselling; SD=standard deviation.

The most common interventions described included CD4 count testing with immediate results (e.g. POC technology) (*n*=4) [21–23,38] , service integration, mainly in the setting of antenatal care (*n*=6) [40,42,45–48], and wider health systems interventions including improved communication, referral and teaching (*n*=4) [37,41,44,49] and incentives (*n*=2) [35,36]. No study provided data on patient satisfaction and costs. Most studies assessed interventions for stage 3 (ART initiation) (*n*=14) and stage 2 (completion of ART eligibility screening) (*n*=9) of the HIV cascade, with only six studies assessing the impact of interventions for stage 1 (linkage to HIV care).

### Impact of interventions

The possible impact of interventions was assessed by comparing proportions of individuals completing staging, enrolling in care, accessing follow-up care or initiating ART in the intervention and control group or by comparing the median time to a positive outcome in individuals receiving the standard of care or the intervention. The definitions of successful outcomes were heterogeneous across studies (Tables 1 and 2) and thus effects of the similar or different interventions were difficult to compare. Studies often reported multiple outcomes along the different steps of the pre-ART period.

**Table 2:** Description of conference abstracts included in the review

Conference	Year of Conference	Author	Year of study	Country	Setting	Group	Study Design	Intervention and Stage	Outcome Measures	Result
19th International AIDS conference	2012	Edmonds [43]	2010–2011	Democratic Republic of Congo	Kinshasa, HIV care and treatment services	Pregnant women	Cluster randomized trial	*Health system:* HIV positive volunteers trained in basic motivational educational techniques who encouraged (and accompanied) women to enrol in HIV treatment and care. Stage 1	Uptake of HIV treatment and care	Cluster adjusted hazard ratio was 1.39 (95% CI 1.01–1.91) comparing intervention vs. control clinics
20th Conference on Retroviruses and Opportunistic Infections	2013	Myer [42]	2011	South Africa	Cape Town, ANC and ART services	Pregnant women	Sequential before/after design	*Behaviour and peer support:* *Enhanced linkage*=PMTCT peer counsellors to accompany ART eligible women from ANC to ART services *Health system:* *Integration*=nurse-midwives provided ART within ANC Stage 3	Initiation of ART before delivery	Pre-intervention: 21% (58/271) Enhanced linkage: 49% (77/157) Integration: 85% (183/214)
19th Conference on Retroviruses and Opportunistic Infections	2012	Solomon [36]	2009–2011	India	Chennai	Injecting drug users	Individual randomized trial	*Incentives* Vouchers that could be redeemed for groceries or household items for attending visits at government ART centres; initiating ART; and viral suppression at 6- and 12-months. Stage 3	Time to ART initiation and number of timely visits to the government centre	Time to initiation: 7 days (intervention) 58 days (control) The median number of timely visits to the government centre was greater 8 (intervention) 3.5 (control)

ART=antiretroviral therapy; CI=confidence interval; PMTCT=prevention of mother to child transmission; ANC=antenatal care.

#### Health system interventions

##### Integration

Integration of ART and antenatal care increased enrolment in HIV care and ART initiation in five out of six observational studies (Tables 1, 2 and 3) [40,42,46–48], with the largest effect observed in a before/after study from Zambia, with pregnant women in the intervention group twice as likely to enrol in HIV care [adjusted odd ratio (aOR)=2.06 (1.27–3.34)] and initiate ART [aOR=2.0 (1.37–2.95)] within 60 days post-diagnosis [47]. An observational study from South Africa examining the impact of integrated ART services within an antenatal care setting showed that pregnant women in the intervention group were less likely to initiate ART compared to the control group [hazard ratio (HR)=0.62 (0.37–1.04)] [45].

**Table 3:** Results (as reported by the authors) of full-text studies included in the review

Authors	Group	Intervention	Outcome: control group	Outcome: intervention group	Unadjusted relative/risk/hazard/odds ratio	Adjusted relative/risk/hazard/odds ratio	Time to outcome: control group, median (IQR)	Time to outcome: intervention group, median (IQR)
**STAGE 1**								
Tsague [40]	Pregnant women	*Health system:* Integration	CD4 count assessment: 111/195 (57%) Enrolling in care: 69/195 (35%)	CD4 count assessment: 547/743 (74%) Enrolling in care: 495/743 (67%)	Relative risk for CD4 count assessment: 1.3 (1.1–1.4) Relative risk for enrolling in care: 1.9 (1.5–2.3)			
Pfeiffer [48]	Unselected and pregnant women	*Health system:* Integration, decentralization			Relative risk for registering with HIV care pregnant women: 2.53 (1.88–3.4)			
Larson [23]	Unselected, tested at a mobile service	*Patient convenience and accessibility:* Immediate CD4 count testing	Linkage to HIV care: 57/122 (47%)	Linkage to HIV care: 117/197 (59%)		Relative risk for linkage to HIV care: 1.23 (1.00–1.57) Absolute risk for linkage to HIV care: 12.7% (1.5–23.9)		
Wanyenze [39]	Inpatients	*Patient convenience and accessibility:* Inpatient testing and counselling, home visits	Linkage to HIV care: 39/249 (16%)	Linkage to HIV care: 52/250 (21%)				
Hatcher [25]	Adults community testing campaign	*Behaviour and peer support:* Peer navigator			HR for enrolment at 10 m for men: 1.48 (1.08–2.04) for women: 1.34 (1.01–1.63)	HR for enrolment at 10 m: for men >25 Y: 1.35 (0.97–1.87) for women: 1.20 (1.00–1.43)		
Killam [47]	Pregnant women	*Health system:* Integration	HIV care enrolment: 181/716 (25%)	HIV care enrolment: 376/846 (44%)	OR for HIV care enrolment 2.36 (1.90–2.94)	OR for HIV care enrolment 2.06 (1.27–3.34)		
**STAGE 2**								
Burtle [49]	Unselected	*Health system:* Comprehensive HIV care	Assessment for ART eligibility: 118/200 (59%)	Assessment for ART eligibility: 152/200 (76%)				
Fairall [51]	Unselected	*Health system:* Task shifting	Enrolment into pre-ART and ART care: 1229/2022 (85%)	Enrolment into pre-ART and ART care: 1229/2022 (85%) 1756/2686 (86%)		Enrolment into pre-ART and ART care: 1.02 (0.92–1.18)		
Jani [21]	Unselected	*Patient convenience and accessibility:* Immediate CD4 count testing	Enrolment into pre-ART and ART care: 1.02 (0.92–1.18)	Completion of staging: 345/437 (79%)	OR for not completing staging: 0.21 (0.15–0.27)	OR for not completing staging: 0.20 (0.15–0.27)	Time to completion of staging: 32 days (19–64) Time between staging and initiation: 5 days (0–19)	Time to completion of staging: 3 days (0–13) Time between staging and initiation: 6 days (0–22)
Faal [22]	Unselected	*Patient convenience and accessibility:* Immediate CD4 count testing	Retention in pre-ART care: 52/139 (37%)	Retention in pre-ART care: 31/81 (38%)	Likelihood for retention in pre-ART care: 1.02 (0.72–1.45)			
Patten [38]	Youth	*Patient convenience and accessibility:* Immediate CD4 count testing	Assessment for ART eligibility: 183/272 (67%)	Assessment for ART eligibility: 275/304 (90%)	Relative risk for assessment of ART eligibility 2.4 ( 1.8–3.4)		Time to eligibility assessment: 36 days	Time to eligibility assessment: 28 days
Muhamadi [52]	Unselected	*Behaviour and peer support:* Extended counselling and peer support	Enrolment into pre-ART care: 77/200 (39%)	Enrolment into pre-ART care: 135/200 (68%)	RR for enrolment in pre-ART care at 5 m: 1.8 (1.4–2.1)			
Kundu [35]	Children	*Incentives:* Food supplement	Regular follow-up 81/100 (81%) Retention in care: 92/100 (92%) Dead and LTFU in pre-ART care: 9/100 (9%)	Regular follow-up in pre-ART care: 74/80 (93%) Retention in pre-ART care: 78/80 (98%) Dead and LTFU in pre-ART care: 3/80 (4%)	HR for irregular follow-up in pre-ART care: 2.89 (1.09–7.63)			
Kohler [50]	Unselected	*Incentives:* Co-trimoxazole with 1 monthly refill	Retention in pre-ART care: 384/610 (63%)	Retention in pre-ART care: 344/414 (83%)		HR for being LTFU in pre-ART care at 12 m: 2.64 (1.95–3.57)		
**STAGE 3**								
Choun [37]	Individuals diagnosed with TB	*Health system*: Integration	ART initiation: 4 weeks: 60/262 (23%) ART initiation: 8 weeks: 170/262 (65%)	ART initiation: 4 weeks: 118/190 (62%) ART initiation: 8 weeks: 175/190 (92%)	HR for ART initiation at 8 weeks: 2.45 (1.79–3.65)	HR for ART initiation at 8 weeks: 2.60 (1.87–3.62)	Time to ART initiation: 6.3 weeks (4.1–10.0)	Time to ART initiation: 3.1 weeks (2.4–5.1)
Tsague [40]	Pregnant women	*Health system:* Integration	ART initiation: 22/26 (85%)	ART initiation: 105/134 (78%)	Relative risk for ART initiation: 0.9 (0.7–1.1)			
Weigel [41]	Pregnant women	*Health system:* Integration	Registration with ART clinic: 2006: 14/53 (26%) 2007: 119/279 (43%) 2008: 101/147 (69%) 2009: 110/133 (83%) ART initiation: 2006: 9/14 (64%) 2007: 94/119 (79%) 2008: 84/101 (83%) 2009: 99/110 (90%)					
Young-leson [44]	Pregnant women	*Health system:* Integration	ART initiation: 124/1243 (10%)	ART initiation: 122/486 (25%)				
Stinson [45]	Pregnant women	*Health system:* Integration	ART initiation: 61/130 (47%)	ART initiation: 124/227 (45%)		HR for ART initiation: 0.62 (0.37–1.04)		
Van der Merwe [46]	Pregnant women	*Health system:* Integration					Time to ART initiation: 56 days (30–103)	Time to ART initiation: 29 days (12–45)
Pfeiffer [48]	Unselected and pregnant women	*Health system:* Integration, decentralization			Relative risk for initiation ART unselected: 1.58 (1.17–2.14)			
Fairall [51]	Unselected	*Health system:* Task shifting	Enrolment into pre-ART and ART care: 1229/2022 (85%)	Enrolment into pre-ART and ART care: 1229/2022 (85%) 1756/2686 (86%)		Relative risk for enrolment into pre-ART and ART care: 1.02 (0.92–1.18)		
Burtle [49]	Unselected	*Health system:* Comprehensive HIV care	ART initiation: 36/68 (53%)	ART initiation: 96/118 (81%)				
Topp [53]	Unselected	*Health system:* Provider initiated testing and counselling	ART initiation: 4523/6520 (69%) Retention in care: 3765/6079 (62%)	ART initiation: 1187/1655 (72%) Retention in care: 1105/1929 (57%)	OR for ART initiation 1.12 (0.99–1.26) OR for retention in care 0.82 (0.74–0.91)	OR for ART initiation 0.90 (0.82–0.97) OR for retention in care 0.84 (0.74–0.95)		
Jani [21]	Unselected	*Patient convenience and accessibility:* Immediate CD4 count testing	ART initiation for eligible patients: 36/93 (39%) ART Initiation for all patients: 57/492 (12%)	ART initiation for eligible patients: 50/144 (35%) ART Initiation for all patients: 94/437 (22%)	OR for ART initiation in eligible patients: 0.84 (0.49–1.45) OR for ART initiation in all patients: 2.05 (1.42–2.96)	OR for ART initiation for eligible patients 2.84 (0.76–10.56)	Time between staging and initiation: 5 days (0–19) Time between testing and initiation: 48 days (34–80)	Time between staging and initiation: 6 days (0–22) Time between testing and initiation: 20 days (10–31)
Faal [22]	Unselected	*Patient convenience and accessibility:* Immediate CD4 count testing	Retention in pre-ART/ART care: 74/220 (34%) ART initiation: 22/71 (31%)	Retention in pre-ART/ART care: 59/124 (48%) ART initiation: 28/43 (65%)	Likelihood for retention in pre-ART/ART care: 1.41 (1.08–1.84) Likelihood for ART initiation: 2.1 (1.39–3.17)			
Patten [38]	Youth	*Patient convenience and accessibility:* Immediate CD4 count testing	ART initiation: 22/48 (44%)	ART initiation: 49/99 (50%)				
Mac-Pherson [26]	Community	*Patient convenience and accessibility:* Home-based ART initiation	ART initiation: 59/8466 (0.7%)	ART initiation: 180/8194 (2.2%)	Risk ratio for ART initiation: 2.94 (2.10–4.12)			

IQR=interquartile range; TB=tuberculosis; HR=hazard ratio; ART=antiretroviral therapy; LTFU=lost to follow-up; OR=odds ratio.

##### Comprehensive HIV care

Three before/after studies investigating packages of health systems interventions aimed at improving referrals, communication and teaching showed an increase in ART initiation by 15% in pregnant women in South Africa [44], by 27% in eight weeks in TB patients in Cambodia [adjusted HR (aHR) for ART initiation within eight weeks post-TB diagnosis=2.60 (1.87–3.62)] [37] and by 15% in unselected adults in Swaziland [49].

##### Task shifting

A *post hoc* analysis of a cluster randomized trial primarily investigating the effect of task-shifting to nurses on mortality found no difference in attrition during pre-ART care six months post-enrolment in South Africa [relative risk (RR)= 1.02 (0.92–1.18)] [51].

##### Provider-initiated testing and counselling

A before/after study in Zambia found that patients who underwent Provider-initiated testing and counselling (PITC) had a significantly lower odds of ART initiation [aOR=0.90 (0.82–0.97)] and pre-ART retention [aOR=0.84 (0.74–0.95)] [53].

#### Patient convenience and accessibility

##### Immediate CD4 count testing

POC CD4 count testing at the time of HIV diagnosis increased the proportion of patients completing an ART eligibility assessment (44% vs. 79%) and reduced time to ART initiation (48 to 20 days *p*<0.0001) [21] in a before/after study in Mozambique. In another study from South Africa, POC CD4 testing doubled ART initiation at healthcare facilities within 60 days post-diagnosis [likelihood ratio=2.01 (1.39–3.17)] and in a randomized controlled trial in South Africa [relative likelihood ratio =2.10 (1.39–3.17)] [21,22]. However, POC CD4 count technology had less of an impact on retention in pre-ART care for individuals not yet eligible for ART [likelihood ratio: 1.02 (0.72–1.45)] [22] and on linkage to HIV services among individuals accessing a mobile HIV testing service [RR=1.23 (1.00–1.57)] [23].

##### Home-based ART initiation

A cluster randomized trial from Malawi showed that optional availability of home-based eligibility assessment following HIV self-testing and ART initiation resulted in a threefold increase in the risk of population-based ART initiation [risk ratio=2.94 (2.10–4.12)] [26].

##### Inpatient testing and counselling, home visits

Similarly, a higher proportion of patients randomized to receive HIV testing as inpatients linked to HIV services (21%) compared to patients who were referred for outpatient HIV testing following discharge from hospital (16%) [39]; however, the result did not reach statistical significance (*p*=0.174).

#### Behaviour interventions and peer support

##### Extended counselling and peer support

A randomized controlled trial conducted in Uganda showed that intensified post-test counselling and monthly visits by a peer support worker almost doubled linkage to HIV care five months post-diagnosis [HR=1.8, (1.42–2.1)] [52]. A peer support intervention among pregnant women in a cluster randomized trial conducted in the Democratic Republic of Congo led to a 39% increase in linkage to HIV care [HR=1.39 (1.01–1.91)] [43], but these results have yet to be formally published.

##### Peer navigator

Visits by peer navigators also increased the likelihood of seeking care following a community-based HIV testing campaign in a observational study conducted in Kenya at 10 months for men (aHR=1.35 (0.97–1.87)] and women [aHR=1.20 (1.00–1.43)] [25]; however, individuals not receiving peer navigator visits were a highly selected group and therefore the results should be interpreted with caution.

#### Incentives

##### Food

Two studies from India investigated the effect of food incentives on retention in pre-ART care and ART initiation. In the first study, the introduction of monthly food supplements increased the proportion of children regularly visiting the clinic over a one year period from 81 to 93% [HR=2.89, (1.09–7.63)]; however, this study was a before/after design and these findings are subject to survival bias as more children were reported dead and lost to follow-up in the control arm (9%) than the intervention arm (4%) [35]. The second study, a randomized controlled trial in injecting drug users, showed that the median time to ART initiation was seven days in individuals receiving food vouchers compared to 58 days in individuals not receiving food vouchers [36].

##### Medical

A third study, from Kenya, found that scheduled and regular visits during the pre-ART period for the purpose of co-trimoxazole refill increased pre-ART retention at 12 months post-enrolment by 20% [50].

### On-going trials

Fifteen on-going or planned trials were identified (Table 4). The majority (*n*=13) of trials are underway in sub-Saharan Africa, six of them in Kenya, and seek to assess interventions for injecting drug users (*n*=3) and pregnant women (*n*=5). Proposed interventions include POC CD4 count testing, rapid (same day) ART initiation, integration, peer educators, intensive counselling, assisted partner notification, home-based male partner testing and short text messages. The majority of trials will be completed by January 2015.

**Table 4:** Description of on-going trials

Title	Status	Country	Subgroup	Intervention	Intervention allocation	Outcomes	Identifier
Linking Infectious and Narcology Care in Russia (LINC) [54]	Study start date: June 2012 Study completion date: April 2016	Russia	Injecting drug users, adults (18+), HIV infected	HIV case management delivered by a peer and point of care CD4 count testing	Individual randomized	Proportion of individuals initiating HIV care within 6 months of enrolment Proportion of individuals retained in HIV care 12 months after enrolment	ClinicalTrials.gov: NCT01612455
An intervention to improve antenatal access to CD4 testing an HAART Botswana [55]	Study start date: July 2011 Study completion date: October 2012	Botswana	Pregnant women, HIV infected	Improved access to CD4 phlebotomy, rapid CD4 result return via SMS messaging sub-Saharan Africa, active follow-up of treatment eligible women, participatory educational sessions for clinic staff, loan program for HIV and CD4 testing supplies	Step-wedge, cluster-randomized	Proportion of eligible pregnant women with CD4 enumeration prior to 26 weeks gestation/prior to delivery Proportion of eligible women with HAART initiation prior to 30 weeks gestation/prior to delivery	ClinicalTrials.gov: NCT01836003
Home-based partner education and testing (HOPE) Study [56]	Study start date: May 2013 Study completion date: June 2014	Kenya	Pregnant women and their partners	Home-based partner education and HIV testing as part of routine pregnancy services	Individual randomized	Proportion of men linkage to HIV care 6 months post-partum (self-report) Proportion of men initiating ART 6 months post-partum (self-report) Proportion of women initiating ART 6 and 14 weeks post enrolment and 6 months postpartum (self-report)	ClinicalTrials.gov: NCT01784783
WelTel Retain: Promoting Engagement in Pre-ART HIV Care Through SMS [57]	Study start date: January 2013 Study completion date: March 2015	Kenya	Adults (18+), HIV infected	Weekly text-messages sub-Saharan Africa	Individual randomized	Proportion of individuals retained in care at 12 months Proportion of individuals completing 1st eligibility assessment within 3 months Proportion of individuals who complete 3 counselling sessions within 2 months	ClinicalTrials.gov: NCT01630304
Testing and linkage to care in injecting drug users in Kenya [58]	Study start date: March 2012 Study completion date: April 2015	Kenya	Injecting drug users, adults (18+)	POC CD4 count and peer care management	Individual randomized	Proportion of individuals linking to care (data collection done in 5 waves separated by 6 months) Time to ART	ClinicalTrials.gov: NCT01557998
Assisted partner notification to augment HIV treatment and prevention in Kenya (APS) [59]	Study start date: June 2012 Study completion date: May 2015	Kenya	Sexual partners of HIV-positive individuals	Assisted-partner notification	Individual randomized	Rate of linkage to HIV care of partners within 6 weeks of index case enrolment	ClinicalTrials.gov: NCT01616420
Mobile phone technology for prevention of mother-to-child transmission of HIV: acceptability, effectiveness and cost [60]	Study start date: May 2011 Study completion date: April 2014	Kenya	Pregnant women, HIV infected	Mobile phone technology	Cluster randomized	Proportion of women who successfully complete key PMTCT transition points from antenatal care to 6 weeks postpartum Proportion of women on ART during labour, delivery and postpartum	ClinicalTrials.gov: NCT01645865
Integration of HIV care and treatment into antenatal care in Migori district, Kenya [61]	Study start date: June 2009 Study completion date: March 2012	Kenya	Pregnant women, HIV infected (18+)	Integrated ANC (health care providers within the ANC department will be trained to provide HIV/PMTCT care)	Cluster randomized	Proportion of women enrolled and retained in HIV care and treatment at 6 months and 1 year	ClinicalTrials.gov: NCT00931216
Optimizing integrated PMTCT services in rural North-Central Nigeria [62]	Study start date: March 2013 Study completion date: September 2014	Nigeria	Pregnant women, HIV infected	Integrated PMTCT package: task-shifting to lower cadre, POC CD4 count testing, prominent role for male partners in collaborations with community health workers	Cluster randomized	Proportion of ART eligible pregnant women who initiated ART for the purposes of PMTCT	ClinicalTrials.gov: NCT01805752
Rapid Initiation of Antiretroviral Therapy to Promote Early HIV/AIDS Treatment in South Africa (RapIT Study) [63]	Study start date: October 2012 Study completion date: May 2015	South Africa	Non pregnant, adults (18+), HIV infected	ART initiation on the day of HIV testing	Individual randomized	Proportion of individuals alive, in care and virally suppressed at 6 months Average time to ART initiation	ClinicalTrials.gov:NCT0171039
Linkage and Retention: A Randomised Trial to Optimize HIV/TB Care in South Africa (Sizanani) [64]	Study start date: August 2010 Study completion date: January 2015	South Africa	Adults (18+), HIV infected	Patient navigator and short message service (SMS), sub-Saharan Africa, contacts throughout the follow-up period	Individual randomized	Proportion of ART eligible individuals linked and retained in ART care 9 months after enrolment	ClinicalTrials.gov: NCT01188941
The “START” (a Streamlined ART Initiation Strategy) Study (START-ART) [65]	Study start date: April 2013 Study completion date: May 2016	Uganda	Adults (18+), HIV infected	POC CD4 testing, targeted knowledge transfer, feedback and reporting to clinic and providers	Randomized roll-out to clinics (step-wedge design)	Cumulative incidence of ART initiation in newly treatment eligible HIV-positive patients	ClinicalTrials.gov: NCT01810289
The PeerCARE Study (Peer Community-based Assistant in Retention) [66]	Study start date: June 2011 Study completion date: June 2014	Uganda	Adults (18+), HIV infected	Peer health worker conducting home visit	Individual randomized	Time to ART initiation within 1 year	ClinicalTrials.gov: NCT01366690
HIV counselling and testing and linkage to care in Uganda^67^	Study start date: May 2008 Study completion date: August 2012	Uganda	Adult (18+) inpatients	More detailed counselling and enhanced linkage to care	Individual randomized(factorial design)	Proportion of individuals receiving of OI prophylaxis and adhering to ART Mortality	ClinicalTrials.gov: NCT00648232
Enhanced access to HIV care for drug users in San Juan, Puerto Rico [68]	Study start date: August 2013 Study completion date: April 2017	Puerto Rico	Injecting drug users (18+)	1) neighbourhood-level HIV testing campaign 2) treatment re-engagement campaign using patient navigators for out of care HIV positive injecting drug users. 3) a patient navigator linkage to care and substance abuse treatment team 4) mobile HIV care clinic to facilitate linkage and retention	Randomized roll-out design (similar to the stepped-wedge design) in 5 neighbourhoods	Proportion of individuals attending HIV care visit at 6 months intervals Proportion of individuals initiating ART at 6 months intervals	ClinicalTrials.gov: NCT017927252

HAART=highly active antiretroviral therapy; SMS=short message service; ART=antiretroviral therapy; POC=point-of-care.

### Quality of studies

Most studies included in this review were observational studies; either times series (before/after studies) (*n*=10) or contemporaneous comparative studies (*n*=7). There were three cluster randomized controlled trials [26,43,51] and four individual randomized trials [36,39,52,22]. Age, sex and CD4 count distributions in control and intervention groups, outcome verification and ascertainment, time delineation of the outcome and controlling for confounding and clustering are described in Table 5. Among the 20 peer-reviewed studies assessed for quality of the evidence, the majority (*n*=8) were rated as having an unclear risk of bias; four studies were classified as being at high risk of bias.

**Table 5:** Quality assessment of published peer reviewed studies

Author	Study design	Selection	Comparability	Outcome	Randomized controlled trials	Summary
Faal [22]	Individual randomized controlled trial				Radom sequence generation – unclear risk of bias Allocation concealment – low risk of bias Blinding of participants – not feasible Blinding of outcome assessment – unclear risk of bias Incomplete outcome data – low risk of bias Selective reporting – low risk of bias Other bias – low risk of bias	Low risk
Fairall [51]	Cluster randomized control trial				Recruitment – low risk of bias Allocation concealment – low risk of bias Statistics – low risk of bias Number of clusters – low risk of bias Outcome – unclear risk of bias (ad-hoc analysis)	Low risk
Muhamadi [52]	Individual randomized controlled trial				Radom sequence generation – unclear risk of bias Allocation concealment – unclear risk of bias Blinding of participants – not feasible Blinding of outcome assessment – unclear risk of bias Incomplete outcome data – low risk of bias Selective reporting – unclear risk of bias Other bias – low risk of bias	Unclear risk
Wanyenze [39]	Individual randomized controlled trial				Radom sequence generation – low risk of bias Allocation concealment – low risk of bias Blinding of participants – not feasible Blinding of outcome assessment – unclear risk of bias Incomplete outcome data – unclear risk of bias Selective reporting – low risk of bias Other bias – unclear risk of bias	Low risk
Hatcher [25]	Observational study with control group	0-0-0	1-0	0-1-0		High risk
Pfeiffer [48]	Observational study with control group	1-0-0	0-0	0-1-0		Unclear risk
Tsague [40]	Observational study with control group	1-1-1	0-0	0-1-0		Unclear risk
Larson [23]	Observational study with control group	1-1-1	1-0	0-1-0		Unclear risk
Stinson [45]	Observational study with control group	1-1-1	1-0	0-1-0		Unclear risk
Topp [53]	Observational study with control group	1-1-1	1-1	0-1-0		Low risk
Choun [37]	Before/after study	1-1-1	1-0	0-1-1		Low risk
Kundu [35]	Before/after study	1-0-1	0-0	0-1-1		High risk
Kohler [50]	Before/after study	1-1-1	1-0	0-1-1		Low risk
Weigel [41]	Before/after study	1-1-1	0-0	0-0-0		Unclear risk
Jani [21]	Before/after study	1-1-1	1-0	0-1-1		Low risk
Youngleson [44]	Before/after study	1-1-1	0-0	0-1-0		Unclear risk
Van der Merwe [46]	Before/after study	1-1-1	0-0	0-1-0		Unclear risk
Burtle [49]	Before/after study	1-1-1	0-0	0-0-0		High risk
Killam [47]	Before/after study	1-1-1	1-0	0-1-0		Low risk
Patten [38]	Before/after study	1-0-1	0-0	0-0-0		High risk

Observational cohort studies:

Selection: studies could score a maximum of 3, the first score for representativeness of the exposed cohort, the second score for the selection of the non-exposed cohort and the third score for ascertainment of exposure.

Comparability: studies could score a maximum of 2, the first score for controlling for important factors, the second score for controlling for any additional factors.

Outcome: studies could score a maximum of 5, the first 2 scores for outcome assessment (a. independent blind assessment, b. record linkage or verification), the second score for time lineated and clear definition of outcome, the last two scores for follow-up (a. complete follow-up, b. subjects with missing outcome assessment unlikely to introduce bias).

Of the four peer-reviewed randomized trials assessed for study quality, the majority were categorized as having low risk of bias (*n*=3) with key quality concerns related to the random sequence generation, allocation concealment and reporting. Of the 16 remaining observational studies, most had an unclear risk of bias (*n*=7) and four studies were rated as having a high risk of bias, with the main quality issues related to uneven distribution of baseline characteristics, no adjustment for confounders, and lack of data related to patient follow-up.

## Discussion

This review identified 24 published studies and 15 on-going trials assessing interventions to improve or facilitate linkage to or retention in pre-ART care and initiation of ART. The majority of these studies were conducted in sub-Saharan Africa and most were observational in design. Patient centred strategies to minimize clinic visits, integration of ART care into antenatal services, education and support for those enrolled in pre-ART care, and incentivized clinic attendance all showed promising results. However, there is a paucity of studies focusing on specific key populations and few studies have investigated a combination of interventions and the impact of these interventions at multiple steps along the pre-ART period. This review suggests that whilst some interventions are specific to certain points along the cascade (e.g. POC CD4 testing for the improvement of linkage to pre-ART care), there are interventions that could potentially support linkage and retention along the entire HIV cascade both in pre-ART and ART care (e.g. integration of HIV services, food incentives, patient navigators) and thus warrants further investigation.

Attending pre-ART clinic visits is a major challenge among patients in low- and middle-income countries due to high transport costs, distance to health care facilities, inability to take time off work, and long clinic waiting times [10] as well as the lack of services (perceived or actual) provided during the pre-ART period [9,10]. Interventions such as POC CD4 testing, home-based ART initiation and integrated service delivery, aimed at decreasing the number of pre-ART visits prior to ART initiation, have proven successful, particularly for those patients immediately eligible for ART. The use of POC CD4 technology or immediate CD4 count testing in South Africa and Mozambique have shown higher completion of ART eligibility assessment, shorter time to staging and ART initiation, and an increase in ART initiations [21–23,38] . Home-based ART initiation is a viable strategy to effectively improve population-level ART initiation as indicated in a trial offering HIV self-testing from Malawi [26]. “Fast tracking” of patients through the provision of counselling and ART eligibility assessment in TB and ANC settings facilitated prompt ART initiation with decreased numbers of clinic visits [37,41]. Results from a randomized controlled trial investigating same day ART initiation on the day of HIV diagnosis, currently underway in South Africa, may provide further evidence on the effect of rapid ART initiation on patient outcomes [63]. However, the long-term effect of reducing the number of clinic visits prior to ART initiation remains unclear. There has been no study to date, except for the Malawian study [26], that has followed-up patients beyond the pre-ART period, and it is plausible that the improvement in linkage and ART initiation is offset by increased default rates once on ART. Given the high HIV burden in this resource-constrained setting, there is a clear need for prioritizing patients based on their baseline CD4 count and urgency to link to care, with more support (e.g. patient navigation, fast-tracking, home-based ART initiation) provided to patients with lower CD4 counts compared to those with higher CD4 counts.

Apart from reducing clinic visit frequency, inflexible clinic hours have been identified as another barrier to accessing care, especially among the working population [10,69,70]. However, none of the included studies investigated the effect of providing services outside traditional clinic operating hours (e.g. evenings and weekends). In addition, interventions targeted at reducing waiting times through increased medical and administrative staffing levels, or appointment-based systems, are yet to be evaluated. A qualitative study from Tanzania investigating referral systems between testing and treatment found that the provision of a transport allowance was a key facilitator to linkage to ART care [24]; however, no study has investigated the impact of transport provision, subsidies, or vouchers on linkage to pre-ART or ART care.

Several studies have investigated the effect of bringing HIV care services closer to where patients reside through decentralization and service integration to overcome issues of distance and difficulties to pay for transport in the context of pre-ART care. A recent systematic review assessing the influence of various models of decentralized HIV treatment and care on initiating and maintaining ART concluded that partial decentralization can decrease attrition of patients on ART [71]. Currently, there is recommendation from recent WHO guidelines to decentralize HIV services from secondary care (e.g. district hospitals) to primary health care clinics [14]. This often goes along with task shifting ART initiation and patient management from doctors to nurses. This is supported by a cluster randomized trial of nurse-led ART initiation in South Africa which showed no detrimental impact on retention in pre-ART care, or immunological and virological outcomes at six months, suggesting that task shifting is a viable approach in some settings [51]. The provision of ART initiation services within or near to ANC clinics as part of a “package” of PMTCT interventions was shown in two studies to increase the proportion of eligible women on ART at delivery [41,44]. In contrast, a positive correlation was seen between distance from the clinic and enrolment for HIV care in an ANC study, suggesting that more distant services were preferred, perhaps due to issues of confidentiality and stigma [25]. These differences are likely to be context specific and therefore this area merits further investigation in different context. Of note, integration of services (i.e. ANC, post-natal and HIV) without clear delineation of the roles, responsibilities and authorities between services could disrupt the continuum of care for women on ART during pregnancy and thus programme planners should consider these requirements prior to implementation.

This review identifies that peer support and appropriate counselling may facilitate linkage to care. Stigma has been identified as a barrier to care at all steps of the HIV cascade, including HIV testing [72], linkage to care [10,73], and ART adherence [74,75]. Fear of disclosure of one’s HIV-positive status is also a reported barrier to care [10], but if achieved, disclosure can support linkage to care [10,76]. Appropriate counselling has been shown to promote disclosure of HIV status to family members, improve adherence to treatment, and promote psychological wellbeing [77–83]. A randomized control trial conducted in Uganda showed that combined post-test counselling by trained staff with home visits by community support agents for on-going counselling also improved linkage to ART services [52]. The costs and feasibility of this labour-intensive approach on a large scale need assessment and further investigation of the effect of different models of post-test counselling on linkage to care is therefore warranted. The use of community-based peer support provided by health care workers and expert patients has been associated with higher rates of ART initiation in the general adult population and ANC settings [43,44]. Several on-going studies will provide evidence into the effect of support systems on ART initiation in high-risk groups such as injecting drug users [54,66,68], pregnant women, and their partners [56]. Mobile phone technologies have been used to trace and support patients once on ART [19,78], and two on-going trials will provide clarity around the effectiveness of the use of mobile technology in pre-ART retention [57,60].

There have been few studies on the use of incentives to improve retention in pre-ART care, including nutritional support, free medications, and conditional cash transfers. Food insecurity was highlighted as a barrier to pre-ART care in a recent systemic review [10] and studies assessing the use of food incentives have shown improved linkage to care in children and injecting drug users [35,36]. Given the potential of improved nutrition to improve treatment outcomes for HIV-positive patients, this area deserves further attention, although costs and feasibility need careful assessment. The provision of free co-trimoxazole was shown to increase retention in pre-ART care for ART-ineligible patients from 63 to 84% in a Kenyan study [50], and further investigation into the cost-effectiveness of medical incentives on retention in pre-ART care are required. Conditional cash transfers have previously been used to promote HIV testing [84–86], and to encourage patients to maintain their HIV-negative status [87,88], but their effect on retention in pre-ART care has not yet been assessed.

This review showed a lack of studies testing interventions aimed at key populations. Men have less access to and coverage of HIV testing and ART in most settings and are at higher risk of attrition from ART and pre-ART services. No study investigating interventions specifically targeted at men were identified [10,89–97]. In addition, despite the high rates of pre-ART attrition amongst children [12] and poor treatment outcomes in adolescents [98–100], only one study was focused on each of these respective groups [35,38]. Ways to support linkage to care for other vulnerable groups such as migrants, prisoners and those in closed settings, sex workers, men who have sex with men, people who inject drugs, and those with HIV-TB co-infection should also be assessed.

Overall, most studies investigated the impact of individual interventions on one stage of the cascade, mainly stages 2 and 3 and few studies have investigated interventions for stage 1, linkage to HIV care.

A single intervention is unlikely to solve attrition across all steps in the pre-ART period and to address the different reasons for attrition. Although, certain interventions such a POC CD4 counting are effective in improving time to linkage to HIV care and completion of ART eligibility screening, it may be a less suitable intervention for improving pre-ART retention, in contrast to food and medical incentives. The effect of interventions targeted at a single point in the pathway might be limited when assessed across the entire pre-ART period. Loss to follow-up prevented earlier on in the pathway might not guarantee retention later on. However, most of the planned or on-going trials investigate single interventions with limited follow-up time. Therefore, a combination of interventions to address the main points at which patient drop-out of care during the pre-ART period or while on ART are most likely to be successful. This package should include interventions aimed at increasing patient convenience and accessibility (e.g. POC CD4 count testing, inpatient testing, home visits and possibly home-based ART initiation) as this was found to be effective in increasing ART eligibility screening and initiation rates; health system interventions such as integration of ART and ANC care as this appears to increase HIV care and ART enrolment; behavioural interventions and peer support (e.g. intensified post-test counselling and peer support) as these were found to increase linkage to care. HIV programmes should first determine the patient groups at risk for drop-out in their cohorts and reasons for drop-out (e.g. too ill to travel to the clinic, disclosure and stigma issues) in order to inform the selection of an effective package of interventions. Programme planners should be mindful of several key factors when selecting interventions i.e.: the operational needs of specific interventions (e.g. staff duties, supplies, data collection systems), challenges in implementing and maintaining interventions (i.e. POC CD4 count testing, integration of care), interventions studied (e.g. incentives) could be context-specific and may be need to be tailored to the target population in their settings. Furthermore, programmes adopting the WHO guidelines supporting earlier ART initiation will likely observe a significant reduction in the number of people with HIV required to remain in the “pre-ART” period, however these programmes will need to implement interventions to improve immediate linkage to ART care as majority of people will require ART care.

The main strengths of this review are the use of a broad search strategy that included an extensive expert consultation, allowing identification of both published and unpublished studies as well as information on on-going trials, a rigorous assessment of study risk of bias and quality. This assessment found that the evidence base and methodology of this current review was subject to several limitations. Firstly, the quality of studies was limited with the majority of studies being observational, using time series rather than concurrent control groups, and subject to unclear risk of bias. Outcome ascertainment was rarely adequately described and time delineation was often lacking, making it difficult to compare outcomes and calculate summary estimates. Assessment of cost-effectiveness and patient acceptability were absent from all studies. Publication bias is likely present as studies with unfavourable results are less likely to be published. In addition, HIV programme evaluation reports from resource-limited settings might not necessarily be published and thus might have been missed by this review. While the literature review was extensively conducted in four international databases including primarily studies published in peer reviewed journals, regional databases such as the African Healthline and LILAC were not searched. The review was limited to low- and middle-income countries. Thus some potential interventions from high income countries which might be applicable to low- and middle-income countries were not included in the review. Most of the studies included were conducted in sub-Saharan Africa, limiting the generalizability of our findings beyond this region and even within the region there is considerable heterogeneity of current service delivery models and approaches. Most studies assessed interventions within the ANC and PMTCT setting and thus findings may not be generalizable to general HIV care programmes. Finally, the majority of studies looked at the effect of a single intervention on a single point in the HIV continuum.

## Conclusions

The overall findings from this review suggest that streamlining services to minimize patient facility visits, providing adequate counselling, medical and peer support, and providing incentives may decrease attrition between HIV testing and ART initiation. Further implementation research, focused programme evaluation and studies with rigorous study designs investigating the impact of individual interventions and a combination of interventions across the HIV cascade, including for the various groups at high risk of attrition, is warranted in order to inform HIV policy and programmes. There is a specific need to also evaluate interventions for key populations as currently they are not only disproportionately affected by HIV but have lower access to and coverage of HIV testing and ART.
